# The Neglected Tropical Diseases and Their Devastating Health and Economic Impact on the Member Nations of the Organisation of the Islamic Conference

**DOI:** 10.1371/journal.pntd.0000539

**Published:** 2009-10-27

**Authors:** Peter J. Hotez

**Affiliations:** 1 Department of Microbiology, Immunology, and Tropical Medicine, George Washington University Medical Center, Washington, D. C., United States of America; 2 Sabin Vaccine Institute, Washington, D. C., United States of America

**Figure 1:**
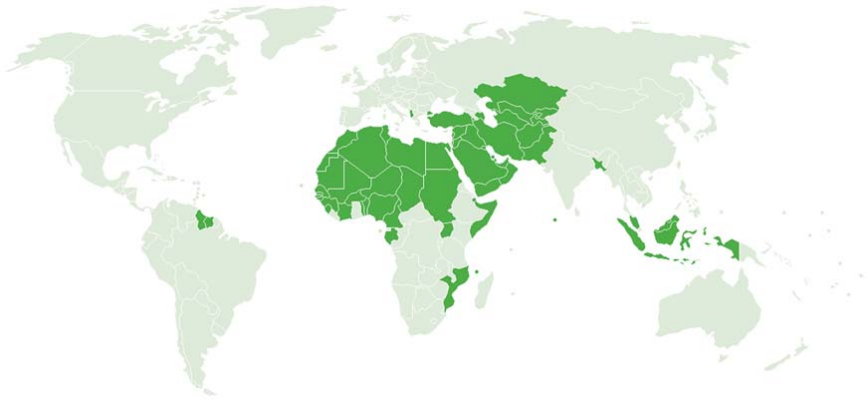
OIC member states. Image credit: Organisation of the Islamic Conference [37].

Founded in 1969, the Organisation of the Islamic Conference (OIC) is comprised of 57 nations that together represent the second largest international organization after the United Nations [1]. According to their Web site, the OIC serves as the “collective voice of the Muslim world,” both protecting its interests and settling conflicts and disputes between member states [1]. In addition to several important and prosperous oil- and gas-producing nations in the Middle East, the OIC nations also include some of the world's poorest countries as well as large middle-income countries with regions of great poverty (Figure 1). In these geographic areas of poverty are also found some of the highest infection rates and endemicity of the neglected tropical diseases (NTDs). Shown in Table 1 is the estimated prevalence of six of the most common NTDs among the most populous OIC member states [2]–[8]. Each of the 28 countries listed has a population that exceeds 10 million people; together they account for more than 90% of the populations living in the OIC.

**Table 1:** The Prevalence of Neglected Tropical Diseases in OIC Member Nations.

Country (Population in 2006^a^)	Ascariasis^b^	Trichuriasis^b^	Hookworm^b^	Schistosomiasis^c^	Leprosy^d^	Trachoma^e^
Indonesia (223 million)	90 million	95 million	62 million	<0.1 million	21,430	-
	42%	44%	28%	<1%		
Pakistan (159 million)	21 million	1.5 million	2 million	-	856	277,560
	14%	1%	1.5%			<1%
Nigeria (145 million)	55 million	34 million	38 million	29 million	5,381	428,065
	45.5%	28%	31%	23%		<1%
Bangladesh (144 million)	78 million	66 million	51 million	-	4,463	-
	55%	46%	35%			
Egypt (75 million)	8 million	2 million	4 million	7 million	1,592	21,654
	12%	2%	5%	10%		<1%
Turkey (72 million)	-	-	-	-	NA	-
Iran (69 million)	5 million	2 million	0.4 million	-	182	15,722
	7.5%	2%	0.5%			<1%
Sudan (37 million)	0.4 million	0.4 million	8 million	5 million	940	3,614,367
	1%	1%	25%	15%		10%
Algeria (33 million)	0.1 million	0.2 million	<0.1 million	2 million	-	143,356
	<1%	1%	<0.1%	8%		<1%
Afghanistan (32 million)	NA	NA	NA	-	32	217,457
						<1%
Morocco (30 million)	1 million	3 million	<0.1 million	<0.1 million	NA	17,134
	4%	11%	<1%	<1%		<1%
Uganda (30 million)	4 million	3 million	9 million	5 million	472	1,050,797
	17%	10%	37%	20%		3%
Iraq (27 million)	1 million	0.2 million	<0.1 million	<0.1 million	-	140,697
	4%	1%	<1%	<1%		<1%
Uzbekistan (27 million)	-	-	-	-	NA	-
Malaysia (26 million)	9 million	12 million	5 million	-	681	-
	38%	49%	19%			
Saudi Arabia (24 million)	0.4 million	0.4 million	0.4 million	<0.1 million	14	-
	2%	2%	2%	<1%		
Yemen (22 million)	6 million	1.5 million	0.1 million	3 million	486	204,984
	30%	8%	1%	15%		1%
Mozambique (20 million)	4 million	6 million	NA	13 million	1,830	390,721
	23%	34%		(70%)		2%
Syria (19 million)	-	-	-	<0.1 million	2	-
				<1%		
Cote d'Ivoire (18 million)	7 million	5 million	10 million	7 million	1,165	601,888
	44%	30%	61%	40%		3%
Cameroon (17 million)	7 million	9 million	3 million	2 million	520	530,102
	46%	57%	19%	12%		3%
Kazakhstan (15 million)	-	-	-	-	NA	-
Niger (14 million)	0.3 million	0.2 million	2 million	3 million	539	2,042,055
	2%	1%	18%	27%		14%
Mali (14 million)	<0.1 million	<0.1 million	2.5 million	8 million	439	1,352,706
	<1%	<1%	20%	60%		10%
Burkina Faso (14 million)	0.1 million	<0.1 million	3 million	8 million	578	1,195,699
	1%	<1%	27%	60%		8.5%
Senegal (12 million)	3 million	2 million	1 million	1.5 million	433	357,191
	28%	19%	9%	15%		3%
Tunisia (10 million)	-	-	-	-	1	-
Chad (10 million)	-	-	3 million	2 million	976	1,016,889
			33%	22.5%		10%
Total OIC Countries (1.34 billion)	300 million	243 million	204 million	95.5 million	43,012	13 million
% People Infected	22%	18%	15%	7%		1%
Percentage of World's Cases in OIC Countries	37%	40%	35%	46%	20%	21%

Only countries with a population of over 10 million are listed. Dashes indicate that these infections are not considered a public health problem in the country. NA, not available.

a Population figures from reference [2], except for Iraq and Afghanistan, which are found in references [3] and [4], respectively.

b Data from reference [5]. Percentage infections based on 2003 population estimates.

c Data from reference [6]. Percentage infections based on 2003 population estimates.

d Data from reference [7].

e Data from reference [8], querying for trachoma in the selected countries.

The information in Table 1 portrays a devastating burden of disease from NTDs in the Islamic world. Unlike better known infections that occur in North America and Europe, the NTDs represent the most common infections of poor people living in developing countries, causing chronic and debilitating conditions that result in impaired childhood growth and developmental delays, poor pregnancy outcome, and reductions in agricultural worker productivity [5],[9],[10]. As a result, the NTDs not only adversely affect health, but they also represent a major reason why poor people living in the OIC and elsewhere cannot escape poverty [9],[10]. For example, between 200 and 300 million people living in OIC countries are infected with one or more intestinal helminth infections, i.e., ascariasis, trichuriasis, and hookworm. Approximately one-half of these cases occur in Indonesia and Bangladesh, two of the most populous OIC countries [5], followed by Nigeria and other African nations [5],[11]. In addition, high rates of intestinal helminth infections occur in Malaysia [12]. Together, the OIC member states account for up to 40% of the global burden of intestinal helminth infections. Children living in the affected countries on average harbor the largest number of intestinal helminths compared to any other age group, and as a result suffer growth stunting, reductions in physical fitness, and developmental and delays [5],[9],[13]. Intestinal helminths impair the ability of a child to learn in school [9],[13], which probably accounts for the observation that chronic hookworm infection in childhood reduces future wage-earning [14]. High rates of hookworm infection also occur during pregnancy, and represent a major cause of anemia among African women [15],[16]. It has been noted that Sahelian nations exhibit higher rates of hookworm infection compared to other intestinal helminthiases, possibly as a result of the high thermal tolerance of hookworm larvae in the soil [15],[17]. This observation likely accounts for the high prevalence rates of hookworm infection in the OIC nations of Burkina Faso, Chad, Mali, Niger, and Sudan.

Schistosomiasis is also a common NTD in the Islamic world. Almost one-half of the world's schistosome infections occur in OIC member states, especially in Nigeria, Mozambique, Burkina Faso, Mali, and Cote d'Ivoire [6]. Many of these cases are urinary tract schistosomiasis caused by *Schistosoma haematobium* [18],[19]. In addition to the end-organ pathology to the bladder, ureters, and kidneys [20], *S. haematobium* infection is associated with reductions in child growth and development similar to those caused by the intestinal helminths [21]. According to some estimates, the disease burden resulting from schistosomiasis may exceed that of malaria [21]. In addition to the intestinal helminth infections and schistosomiasis, both lymphatic filariasis and onchocerciasis are highly prevalent NTDs in the OIC member countries. The bacterial NTDs are also prominent. Approximately 20% of the world's 213,000 registered cases of leprosy [7] and 21% of the world's cases of blinding trachoma [8] occur in OIC countries. Blinding trachoma exhibits the highest prevalence in the Sahelian countries of Sudan (almost 4 million cases), Niger (2 million), and Burkina Faso and Mali (1 million cases each) [8], where the dry and dusty conditions there combine with extreme poverty, inadequate sanitation, and poor access to clean water to ensure high rates of transmission [22]. In Sudan, blinding trachoma has also emerged in the setting of conflict [23].

Indeed, several conflict and post-conflict countries in the OIC stand out for their high prevalence rates of NTDs. In Sudan, the high endemicity of trachoma, hookworm and other intestinal helminth infections, and schistosomiasis were already mentioned. In addition, dracunculiasis (guinea worm) is still prevalent, and more cases of guinea worm occur there relative to any other country [11],[24]. Some of the world's highest rates of visceral leishmaniasis also occur in Sudan, particularly along the border with Ethiopia where refugees living under conditions of extreme stress are exposed to sandfly vectors [11],[25],[26]. For similar reasons, visceral leishmaniasis is endemic to Somalia, where extremely high rates of schistosomiasis are also present [6],[26]. War-torn areas of Afghanistan (and much of Pakistan) and Iraq exhibit a high prevalence of both leishmaniasis (especially cutaneous leishmaniasis) and ascariasis [27]–[29]. Unlike other intestinal helminths, the eggs of *Ascaris lumbricoides* are capable of withstanding the cold and dry conditions that can occur in these countries. Ascariasis and other intestinal parasitic infections are also highly prevalent among children living in the Gaza strip [30]. I have suggested previously that the NTDs not only emerge in the setting of conflict, but these infections may also promote conflict through their destabilizing effects on human populations and agriculture [31].

The health and socioeconomic effects of the NTDs, including their poverty-promoting and conflict-promoting features, should provide strong incentives to try and control or eliminate these infections in the poorest countries of the OIC. Indeed, through mass drug administration with either low-cost generic drugs or drugs donated by multinational pharmaceutical companies, most of the NTDs listed in Table 1, in addition to lymphatic filariasis and onchocerciasis, can either be controlled or in some cases eliminated at extremely low costs [32],[33]. For instance, in Africa, a package of drugs that simultaneously target the intestinal helminth infections, schistosomiasis, trachoma, lymphatic filariasis, and onchcerciasis can be administered on an annual basis for as little as US$0.50 per person [9],[32],[33], while leprosy elimination may be feasible through case detection and multi-drug therapy, also with donated drugs [10]. Therefore, large-scale control programs for some of the most prevalent and poverty-promoting NTDs could be implemented in the endemic OIC countries for a fraction of the costs required for better known conditions such as HIV/AIDS, malaria, or tuberculosis, and far less than needed for non-infectious chronic diseases [9],[10],[32],[33].

Through private donations from the Bill & Melinda Gates Foundation and other foundations, the United States government, the British Department for International Development, and the World Health Organization are working together with public–private partnerships aligned through the Global Network for Neglected Tropical Diseases and several non-governmental organizations, including the Carter Center, to implement and integrate mass drug administration for the most common NTDs in more than one dozen endemic countries [9],[33]. Given the health and economic importance of NTDs in the OIC member states, it is also appropriate to look to some of the prosperous Arab nations, including Saudi Arabia, Kuwait, the United Arab Emirates (UAE), Qatar, and Bahrain, for additional funds to support NTD control. Recently, through support from the Gates Foundation, the Global Network for Neglected Tropical Diseases is establishing regional nodes in Africa, Asia, and the Americas that could offer appropriate mechanisms to channel funds from either prominent families or governments from some of these Middle Eastern countries.

The impressive establishment of new and distinguished universities in Saudi Arabia, UAE, Qatar, and elsewhere [34],[35] could also be tapped to provide training in tropical diseases. Currently, no school of tropical medicine exists in the Middle East, i.e., one which is similar to either the Liverpool School of Tropical Medicine or the London School of Hygiene and Tropical Medicine [36]. Establishment of such an institution in the Persian Gulf region in order to specifically target health disparities in the OIC countries would represent a breakthrough in medical and public health education in the Middle East. A comprehensive assault on NTDs represents one of the most cost-efficient mechanisms to improve health among the poorest people living in OIC countries and to simultaneously lift them out of poverty [9]. NTD control may also serve to reduce tensions and conflicts in highly endemic OIC countries [31]. Joint action between the G8 countries and prominent families and governments in the Persian Gulf, together with technical assistance by WHO and financial mechanisms of the Global Network for Neglected Tropical Diseases would represent an impressive beginning.
